# CDH2 gene rs11564299 polymorphism is a risk factor for knee osteoarthritis in a Chinese population: a case–control study

**DOI:** 10.1186/s13018-019-1256-0

**Published:** 2019-07-09

**Authors:** Houlai Shang, Yuedong Hao, Wenhao Hu, Xiaohui Hu, Qing Jin

**Affiliations:** 1 Department of Orthopaedics, The Affiliated Huaian No.1 People’s Hospital of Nanjing Medical University, Huai’an, 223300 Jiangsu China; 2 Department of Operation and Anesthesiology, The Affiliated Huaian No.1 People’s Hospital of Nanjing Medical University, Huai’an, 223300 Jiangsu China

**Keywords:** CDH2, Knee osteoarthritis, Single nucleotide polymorphism (SNP)

## Abstract

**Background:**

Cadherin-2 (CDH2) gene polymorphisms were reported to be associated with the induction and development of knee osteoarthritis (OA).

**Methods:**

This case–control study was designed to explore the association between CDH2 gene rs11564299 polymorphism and the risk of knee OA in Chinese subjects. The polymorphism was genotyped by polymerase chain reaction and Sanger sequencing.

**Results:**

G allele or GG genotype of CDH2 gene rs11564299 polymorphism was related to increased risk for knee OA in the Chinese Han population. Additionally, subgroup analyses indicated that the female, smoker, drinker, and BMI ≥ 25 kg/m^2^ groups showed increased risk for knee OA. Additionally, this polymorphism was associated with CRP and Kellgren–Lawrence grade.

**Conclusion:**

In summary, this current study reveals that CDH2 gene rs11564299 polymorphism is a risk factor for knee OA development in this Chinese population. The genotypes distribution differed significantly among OA patients and healthy controls and may be a useful tool in the evaluation of OA susceptibility in Chinese Han population.

## Background

Osteoarthritis (OA) is one of the most common chronic joint diseases. It leads to diminished quality of life and even disability in the elderly worldwide. The main characteristic of OA is pain, limitation, and dysfunction of joint activity [1], and a surgical treatment is often necessary. The radiographic definition for OA is according to the criteria of Kellgren–Lawrence (K/L) radiographic grading scheme. This overall joint scoring system grades OA from 0 to 4 score, which defines OA by the presence of a definite osteophyte (grade ≥ 2), and the presumed successive appearance of joint space narrowing, cysts, sclerosis, and deformity were regarded as more severe grades. The underlying pathophysiological mechanisms of OA primarily involve subchondral bone remodeling, synovial inflammation, osteophyte formation, ligamentous laxity, and the weakening of periarticular muscles. OA accounts for more difficulty with walking and climbing stairs than any other disorders [2]. OA is also the most common cause for total hip and total knee replacement [3]. OA shows a growing impact on health care and public health systems in China. Among all the sites, knee OA takes the highest percent, affecting up to 6% of all adults [4]. As the life expectancy of the Chinese population increases, the morbidity of knee OA is rising every year. Besides increased age, certain factors such as trauma, joint injuries, overweight, and physical activity may result in the development of knee OA [5, 6]. Biomechanical, molecular, and morphological changes of cells and extracellular matrix contribute to a progressive cartilage loss, osteophyte formation, and synovitis as well as degeneration of the joint [7]. An increasing body of evidence suggested that multiple genetic factors affect the pathogenesis of several kinds of OA [8–10]. Previous studies have shown that OA is inherited and may vary by joint site. Recently, studies showed many candidate genes including Cadherin 2 (CDH2) as susceptibility loci for OA in different ethnic groups.

CDH2, also known as Cadherin 2 or N-cadherin, is a member of the cadherin gene superfamily. Cadherins are a group of cell surface molecules which recognize direct intercellular contact and mediate signal pathways into the cells. CDH2 is a kind of glycoproteins, which has been proved to play an important role in Ca2+ attachment and regulation of cell–cell functional protein adhesion [11, 12]. CDH2 was reported to have vital impacts on neurological diseases such as obsessive–compulsive disorder [13] and epilepsy [14]. Additionally, it was involved in tumor progression and resistance [15–17] because it worked as an intercellular signal mediator. In previous studies, CDH2 gene rs11564299 polymorphism seemed to take part in the development and risk of OA [18, 19], but the conclusions drawn by these studies were inconsistent. Therefore, we designed this case–control study to validate the association between CDH2 gene rs11564299 polymorphism and the risk of knee OA in a Chinese Han population.

## Methods

### Patients

We recruited 348 OA cases and 423 control consecutively from the Huai’an First People’s Hospital. Patients were diagnosed on the basis of the American College of Rheumatology criteria, which included primary OA with Kellgren–Lawrence grade (K-L grade) ≥ 2. The exclusion criteria were as follows: inflammatory arthritis, post-traumatic, any type of cancer, or developmental dysplasia. Participants were chosen without restriction to age, sex, or occupation.

The healthy controls were selected from subjects receiving regular medical examination. No controls ever had any clinical or symptom sign of OA, other arthritis, or joint diseases (pain, swelling, tenderness, or restriction of movement) at any site based on medical record. No controls had relation to the patients or family history of OA.

The demographic, lifestyle, and clinical characteristics of all participants, such as gender, age, body mass index (BMI), erythrocyte sedimentation rate (ESR), C-reactive protein (CRP), and K-L grading, were collected from medical records. Individuals who had smoked at least 1 cigarette per day more than 1 year were considered to be smoker. We defined alcohol usage as consumption of at least one drink per week. Written informed consent was obtained from all participants prior to their participation. The research protocol was approved by the Ethics Committee of The Affiliated Huaian No.1 People’s Hospital of Nanjing Medical University. The ethical approval was consistent with the standards of the Declaration of Helsinki.

### DNA extraction and genotyping

Two milliliters of peripheral blood samples were collected using vacutainer tubes and transferred to tubes containing EDTA and stored at 4 °C until use. Genomic DNA was extracted using a QIAamp DNA blood mini kit (Qiagen, Hilden, Germany) following the manufacturer’s instruction. The integrity and purity of the extracted DNA were tested by measuring the absorbance and running electrophoresis.

Genotyping was carried out by polymerase chain reaction (PCR) and Sanger sequencing method. The primers used for the nucleotide extension reaction were 5′-AATAATAAATAATAGGCCTCTGATTACAAGA-3′ and 5′-ATAATAAATAATAGGCCTCTGATTACAAGG-3′. PCRs were performed each in a 50-μL volume containing 1 μL of DNA template, 5 μL of 10× PCR buffer containing 1.5 μM MgCl_2_, 1 μL of 1 U/μL Taq DNA polymerase, 5 μL of 2.5 mM dNTPs, 33 μL of sterilized distilled water, and 1 μL of forward/reverse primers. The amplification conditions were as follows: 95 °C for 10 min; 30 cycles of 94 °C for 30 s, 55 °C for 30 s, 72 °C for 30 s; and final extension for 10 min at 72 °C. The PCR products were electrophoresed on 2% agarose gel and subjected to direct sequencing. In order to control the genotyping quality, 3% of samples were randomly selected for repeated assay by two different persons.

### Statistical analysis

The chi-square test was applied for categorical data, and unpaired Student’s *t* test and analysis of variance (ANOVA) were used for continuous data. A goodness-of-fit chi-square was used to test the Hardy–Weinberg equilibrium (HWE) of genotype distributions among controls. Odds ratios (ORs) and 95% confidence intervals (CIs) were calculated to evaluate the OA risk associated with genotypes and alleles of rs11564299 polymorphism by logistic regression analyses. All statistical analyses were performed using SAS 9.1.3 (SAS Institute, Cary, NC, USA). *P* < 0.05 was the cutoff value of significance.

## Results

### Characteristics of the study population

No significant difference between the patients and the controls was found in the mean age (62.23 ± 8.13 years vs. 62.50 ± 7.64 years, *P* = 0.640) or the male/female ratio (69/270 vs. 75/348, *P* = 0.457) (Table 1). There was no significant between-group difference in terms of BMI. Similarly, the proportions of smokers or drinkers did not differ significantly. K-L grade II+III accounted for the majority of OA patients.

**Table 1:** Patient demographics and risk factors in osteoarthritis

Variable	Cases (*n* = 348)	Controls (*n* = 423)	*P*
Age (years)	62.23 ± 8.13	62.50 ± 7.64	0.640
Body mass index, kg/m^2^	24.37 ± 1.55	24.27 ± 1.47	0.327
Sex			0.457
Male	69 (19.83%)	75 (17.73%)	
Female	279 (80.17%)	348 (82.27%)	
Smoking			0.885
Yes	102 (29.31%)	126 (29.79%)	
No	246 (70.69%)	297 (70.21%)	
Alcohol			0.710
Yes	132 (37.93%)	166 (39.24%)	
No	216 (62.07%)	257 (60.76%)	
ESR, mm/h	10.64 ± 11.43		
CRP, mg/L	7.69 ± 17.47		
Kellgren–Lawrence grading			
II	180 (51.72%)		
III	102 (29.31%)		
IV	66 (18.97%)		

*CRP* C-reactive protein, *ESR* erythrocyte sedimentation rate

### Association between CDH2 rs11564299 polymorphism and OA risk

The genotype and allele distributions for rs11564299 polymorphism are shown in Table 2. Genotype distributions of these polymorphisms in the controls conformed to HWE (*P* = 0.798). Logistic regression analyses revealed the risk of OA was significantly intensified by GG genotype (OR and 95%CI 2.66(1.30–5.47), *P* = 0.008 for GG vs. AA; 1.47(1.09–1.98), *P* = 0.013 for AG+GG vs. AA) or G allele carriers (1.33 (1.07, 1.63), *P* = 0.008 for A vs. G). Furthermore, we have investigated the role of CDH2 rs11564299 polymorphism in the risk of OA stratified by age, sex, smoking, BMI, and drinking (Table 3). The association between CDH2 rs11564299 polymorphism and OA appeared stronger in the subgroups of females, smokers, drinkers, and subjects with BMI ≥ 25 kg/m^2^.

**Table 2:** Logistic regression analysis of associations between polymorphisms and risk of osteoarthritis

Genotype	Cases* (*n* = 348)		Controls* (*n* = 423)		OR (95% CI)	*P*	OR** (95% CI)	P**
	*n*	%	*n*	%				
rs11564299 A/G								
AA	213	61.6	296	70.1	1.00		1.00	
AG	110	31.8	114	27.0	1.34 (0.98–1.84)	0.069	1.34 (0.98–1.84)	0.071
GG	23	6.6	12	2.8	*2.66 (1.30–5.47)*	*0.008*	*2.68 (1.30–5.51)*	*0.007*
AG+GG	133	38.4	126	29.9	*1.47 (1.09–1.98)*	*0.013*	*1.47 (1.09–1.98)*	*0.013*
AA+AG	323	93.4	410	97.2	1.00		1.00	
GG	23	6.6	12	2.8	*2.43 (1.19–4.96)*	*0.015*	*2.45 (1.20–5.00)*	*0.014*
A allele	536	77.5	706	83.6	1.00		1.00	
G allele	156	22.5	138	16.4	*1.49 (1.15–1.92)*	*0.002*	–	–

Values in italics are statistically significant (*P* < 0.05)

*The genotyping was successful in 346 cases and 422 controls for rs11564299

**Adjusted for sex and age

**Table 3:** Stratified analyses between rs11564299 polymorphisms and the risk of osteoarthritis

Variable	rs11564299 (case/control)			AG vs. AA	GG vs. AA	GG vs. AA+AG	GG+AG vs. AA
	AA	AG	GG				
Sex							
Male	44/50	19/24	6/24	0.90 (0.44–1.86); 0.775	6.82 (0.79–58.80); 0.081	7.04 (0.83–60.06); 0.074	1.14 (0.57–2.26); 0.715
Female	168/246	91/90	17/90	*1.47 (1.04–2.09); 0.031*	*2.25 (1.03-4.92); 0.043*	2.00 (0.92–4.34); 0.080	*1.56 (1.11–2.18); 0.010*
Smoking							
Yes	135/213	79/73	12/7	*1.71 (1.16–2.51); 0.006*	*2.71 (1.04–7.04); 0.042*	*2.29 (0.89–5.92); 0.087*	*1.80 (1.24–2.60); 0.002*
No	78/83	31/41	11/5	0.81 (0.46–1.41); 0.446	2.34 (0.78–7.04); 0.130	2.50 (0.84–7.43); 0.099	0.97 (0.58–1.63); 0.914
Alcohol							
Yes	129/198	71/80	16/6	1.36 (0.92–2.01); 0.119	*4.09 (1.56–10.73); 0.004*	*3.71 (1.43–9.63); 0.007*	*1.55 (1.07–2.25); 0.020*
No	84/98	39/34	7/6	1.34 (0.78–2.31); 0.294	1.36 (0.44–4.21); 0.592	1.25 (0.41–3.83); 0.694	1.34 (0.80–2.24); 0.263
Age (years)							
< 60	77/109	47/38	6/4	*1.75 (1.04–2.94); 0.034*	2.12 (0.58–7.78); 0.256	1.78 (0.49–6.44); 0.381	*1.79 (1.08–2.94); 0.023*
≥ 60	136/187	63/75	17/8	1.14 (0.76–1.70); 0.522	*2.92 (1.23–6.97); 0.016*	*2.81 (1.19–6.64); 0.019*	1.31 (0.90–1.91); 0.162
BMI							
< 25	75/94	43/44	6/4	1.23 (0.73–2.06); 0.443	1.88 (0.51–6.91); 0.342	1.75 (0.48–6.37); 0.392	1.28 (0.78–2.11); 0.335
≥ 25	138/202	67/70	17/8	1.40 (0.94–2.09); 0.098	*3.11 (1.31–7.41); 0.010*	*2.82 (1.19–6.66); 0.018*	*1.58 (1.08–2.30); 0.018*

Values in italics are statistically significant (*P* < 0.05)

Subsequently, we examined the association between rs11564299 polymorphism and clinical features of OA patients, including ESR, CRP, and K-L grade (Table 4). Our results indicated that AG, GG, and AG+GG genotype were more frequent in CRP ≥ 25 mg/L compared to CRP < 25 mg/L and in patients with K-L grade III or IV compared to K-L grade II. This indicated that CDH2 rs11564299 polymorphism was associated with CRP and K-L grade.

**Table 4:** The associations between CDH2 rs11564299 polymorphism and clinical characteristics of osteoarthritis

Characteristics	Genotype distributions			
rs11564299	AA	AG	GG	AG+GG
ESR				
≥ 10/< 10	88/125	43/67	6/17	49/84
OR (95%CI); *P* value	1.0 (reference)	0.91 (0.57–1.46); 0.700	0.50 (0.19–1.32); 0.156	0.83 (0.53–1.29); 0.408
CRP				
≥ 25/< 25	14/199	15/95	5/18	20/113
OR (95%CI); *P* value	1.0 (reference)	*2.24 (1.04–4.84); 0.035*	*3.95 (1.28–12.22); 0.011*	*2.52 (1.22–5.17); 0.010*
K-L grade				
III+IV/II	103/110	70/40	17/6	87/46
OR (95%CI); *P* value	1.0 (reference)	*1.87 (1.17–3.00); 0.009*	*3.03 (1.15–7.97); 0.020*	*2.02 (1.29–3.16); 0.002*

Values in italics are statistically significant (*P* < 0.05).

## Discussion

We evaluated the effects of CDH2 gene rs11564299 polymorphism on knee OA risk in this current Chinese case–control study and found that CDH2 gene rs11564299 polymorphism may work as a risk factor in the progression of knee OA in this Chinese Han population, especially among female, smokers, drinkers, and subjects with BMI ≥ 25 kg/m^2^. This SNP also showed significant correlation with CRP and K-L grade.

Up to date, the etiologies of OA are still poorly understood, which should be attributed to interactions between systemic and local factors [20]. Systemic risk factors involve old age, overweight and obesity, female gender, ethnicity, and genetic factors; local risk factors contain muscle weakness, repetitive use of joints, previous knee injury, bone density, and joint laxity [21]. In this study, we primarily focused on the association between genetic factors and knee OA risk. We chose the CDH2 as the investigate gene.

N-cadherin, encoded by CDH2 gene in the human body, was known as a kind of transmembrane proteins expressed in various tissues and mediates cell–cell adhesion [22, 23]. Changes with the expression and integrity of CDH2 have been observed in various forms of diseases [24, 25]. In addition, it has been reported that high expression of CDH2 in cytoplasm can lead to a remarkable increase of massive oncogenes. Expression of CDH2 has prognostic and predictable significance in kinds of tumors [23, 26]. The change with CDH2 expression may lead to apoptosis of cells in previous studies [27, 28].

Ruedel et al. evaluated the relationship between CDH2 gene rs11564299 polymorphism and OA risk in a Germany population with 312 OA patients and 259 controls and found the minor allele of rs11564299 polymorphism was related to decreased risk for OA [18]. In addition, they showed that compared with carriers of the major allele, minor allele of rs11564299 polymorphism elevated CDH2 expression in synovial fibroblasts [18]. Another study from China by Zhao et al. revealed that rs11564299 polymorphism showed no correlation with OA in a Chinese population [19]. In this study, we found that G allele or GG genotype of CDH2 gene rs11564299 polymorphism was associated with increased risk for knee OA in a Chinese Han population. It is obvious that the conclusions of this study were different from those of another Chinese study. We assumed the following factors may explain it. One, genetic heterogeneity for OA existed in different regions of China. Two, clinical heterogeneity may also be a potential factor. For example, the study by Zhao et al. investigated mixed OA including knee OA and hand OA, while this study only explored knee OA. Three, the sample sizes varied among these studies. Four, diverse lifestyles and BMIs may also contribute to it. Subgroup analysis indicated that individuals who were female, smokers, and drinkers and subjects with BMI ≥ 25 kg/m2 were prone to knee OA in this study. These results should be validated in more well-designed studies considering the relatively limited sample sizes of subgroup analyses.

There were some limitations in this present study. First, the correlation between the CDH2 gene rs11564299 polymorphism and knee OA susceptibility could not be entirely demonstrated by a single case–control study. Second, the facticity might be underpowered because of the relatively small sample size. Third, the patients or the healthy controls were all selected from hospitals, which may cause bias of population representativeness. Fourth, the ethnic distinction could not be ignored since we only designed this study among the Chinese population. And the results of this study cannot be extrapolated to different ethnic groups. Fifth, knee OA is a multifactorial etiopathogenetic disease, and the relationship between this disorder and other environmental factors should be investigated. In addition, the difference of two knee joints, strictly speaking, should be taken into consideration but we did not.

To sum up, CDH2 gene rs11564299 polymorphism is associated with increased risk for knee OA in this Chinese Han population. This SNP may be a potential genetic marker to OA susceptibility in Chinese Han population, which was beneficial to early detection and treatment of OA. More studies with larger sample sizes in other races should be conformed to verify the relationship between this SNP and OA risk.

## Data Availability

Please contact the authors for reasonable requests.

## Abbreviations

BMI: Body mass index
CDH2: Cadherin-2
CIs: Confidence intervals
CRP: C-reactive protein
ESR: Erythrocyte sedimentation rate
HWE: Hardy–Weinberg equilibrium
K-L grade: Kellgren–Lawrence grade
OA: Osteoarthritis
ORs: Odds ratios
SNP: Single nucleotide polymorphism
